# Short- and Long-Term Effects of High-Intensity Interval Training vs. Moderate-Intensity Continuous Training on Left Ventricular Remodeling in Patients Early After ST-Segment Elevation Myocardial Infarction—The HIIT-EARLY Randomized Controlled Trial

**DOI:** 10.3389/fcvm.2022.869501

**Published:** 2022-06-17

**Authors:** Prisca Eser, Lukas D. Trachsel, Thimo Marcin, David Herzig, Irina Freiburghaus, Stefano De Marchi, Andreas J. Zimmermann, Jean-Paul Schmid, Matthias Wilhelm

**Affiliations:** ^1^Department of Cardiology, Cardiovascular Centre, Inselspital, Bern University Hospital, University of Bern, Bern, Switzerland; ^2^Research Department, Berner Reha Zentrum, Heiligenschwendi, Switzerland; ^3^University Clinic for Diabetes, Endocrinology, Nutritional Medicine, and Metabolism, Inselspital, Bern University Hospital, University of Bern, Bern, Switzerland; ^4^Department of Cardiology, Clinic Gais, Gais, Switzerland

**Keywords:** left ventricular end-diastolic volume, global longitudinal strain, cardiac remodeling, exercise training modality, cardiac rehabilitation

## Abstract

**Aim:**

Due to insufficient evidence on the safety and effectiveness of high-intensity interval training (HIIT) in patients early after ST-segment elevation myocardial infarction (STEMI), we aimed to compare short- and long-term effects of randomized HIIT or moderate-intensity continuous training (MICT) on markers of left ventricular (LV) remodeling in STEMI patients receiving optimal guideline-directed medical therapy (GDMT).

**Materials and Methods:**

Patients after STEMI (<4 weeks) enrolled in a 12-week cardiac rehabilitation (CR) program were recruited for this randomized controlled trial (NCT02627586). During a 3-week run-in period with three weekly MICT sessions, GDMT was up-titrated. Then, the patients were randomized to HIIT or isocaloric MICT for 9 weeks. Echocardiography and cardiopulmonary exercise tests were performed after run-in (3 weeks), end of CR (12 weeks), and at 1-year follow-up. The primary outcome was LV end-diastolic volume index (LVEDVi) at the end of CR. Secondary outcomes were LV global longitudinal strain (GLS) and cardiopulmonary fitness.

**Results:**

Seventy-three male patients were included, with the time between STEMI and start of CR and randomization being 12.5 ± 6.3 and 45.8 ± 10.8 days, respectively. Mixed models revealed no significant group × time interaction for LVEDVi at the end of CR (*p* = 0.557). However, there was a significantly smaller improvement in GLS at 1-year follow-up in the HIIT compared to the MICT group (*p* = 0.031 for group × time interaction). Cardiorespiratory fitness improved significantly from a median value of 26.5 (1st quartile 24.4; 3rd quartile 1.1) ml/kg/min at randomization in the HIIT and 27.7 (23.9; 31.6) ml/kg/min in the MICT group to 29.6 (25.3; 32.2) and 29.9 (26.1; 34.9) ml/kg/min at the end of CR and to 29.0 (26.6; 33.3) and 30.6 (26.0; 33.8) ml/kg/min at 1 year follow-up in HIIT and MICT patients, respectively, with no significant group × time interactions (*p* = 0.138 and 0.317).

**Conclusion:**

In optimally treated patients early after STEMI, HIIT was not different from isocaloric MICT with regard to short-term effects on LVEDVi and cardiorespiratory fitness. The worsening in GLS at 1 year in the HIIT group deserves further investigation, as early HIIT may offset the beneficial effects of GDMT on LV remodeling in the long term.

## Introduction

Despite advances in treatment strategies for myocardial infarction (MI) in recent decades, left ventricular (LV) dysfunction and heart failure may develop in up to one-third of all MI patients and are associated with a high risk of subsequent death or heart failure readmission (1).

In combination with guideline-directed medical therapy (GDMT), aerobic exercise training has been shown to potentially reverse cardiac remodeling after recent (<3 months) MI, with more beneficial effects when exercise is started soon after MI rather than later, and with longer compared to shorter exercise program durations (2, 3). Several studies and meta-analyses in coronary artery disease (CAD) from the last decades have indicated that high-intensity interval training (HIIT) may be superior to moderate-intensity continuous exercise (MICT) in improving peak oxygen consumption (VO_2_) (4–6). However, studies comparing isocaloric training modalities found short-term improvements in peak VO_2_ to be comparable between HIIT and MICT (6). Only a few studies have investigated the effects of different exercise modalities on LV reverse remodeling in MI patients. One small single-center study in chronic ischemic heart failure patients has indicated that reverse LV remodeling may be superior with HIIT as compared to MICT (7); however, this could not be confirmed by a larger multi-center study (8). Another recent study included patients within 30 days after acute coronary syndrome and found better LV function echocardiographically in patients after HIIT compared to MICT training; however, baseline values and changes from baseline were not indicated (9). In the SAINTEX-CAD study including 200 patients with chronic coronary syndromes (58% with a history of acute MI), a trend for a more favorable reverse remodeling was found with MICT compared to the HIIT intervention (10).

While short-term safety with regard to cardiac events within the 4 h following HIIT sessions in CAD patients has been found to be reasonable (11), long-term safety regarding LV remodeling has not been addressed. Based on the paucity of data on LV remodeling with different exercise modalities in MI patients, the European Association of Preventive Cardiology endorsed HIIT only for low-risk patients (12). A concern was the safety and feasibility of HIIT in acute coronary syndrome (ACS) patients. Only a few studies comparing HIIT with MICT reported the inclusion of patients early after MI, and their results with regard to improvements in peak VO_2_ were controversial (13–16). Moholdt et al. (15) as well as Keteyian et al. (14) found a significantly greater increase in peak VO_2_ with HIIT than with MICT, however, their mean duration until training started after MI was 50 and approximately 30 days, respectively, hence they included only a few patients early after MI. In the pilot study by Trachsel et al. (17) they also found a greater increase in peak VO_2_ with HIIT; however, they did not specify the time after MI other than that the maximal duration after MI for study inclusion was 6 weeks. No difference between improvements with HIIT and MICT was found in the large study by Conraads et al. (13) who included patients within 4–12 weeks after acute MI, PCI, or coronary artery bypass grafting, without indicating mean duration after MI. We have recently shown that self-tailored HIIT is feasible in patients early (within 4 weeks) after ST-segment elevation MI (STEMI), and equally effective with regard to peak VO_2_, compared to isocaloric MICT (18). Effects of HIIT on LV remodeling in patients after acute MI have only been investigated by one large study including 75 patients within 30 days from complete revascularization by the percutaneous coronary intervention (PCI) after ACS (9), and two smaller studies, one including patients after PCI and coronary artery bypass grafting with a longer mean inclusion time of 74 days after MI (19) and the other performing HIIT in only nine patients (17). All of these studies indicated that HIIT after recent ACS leads to favorable LV remodeling; however, comparison with LV remodeling after MICT was incompletely performed. D’Andrea et al. (9) did not present changes from baseline values, and Lund et al. (19) did not include a MICT group. Neither of these studies included only STEMI patients, and neither of them included a long-term follow-up of LV remodeling.

The aim of the present study was to investigate the short- and long-term safety and effectiveness of HIIT compared to MICT in high-risk ACS patients, namely in patients early after an acute STEMI. Based on the promising results of earlier studies (9, 10, 17, 19), we hypothesized that short- and long-term effects of 9 weeks of HIIT on markers of LV remodeling, namely LV end-diastolic volume index (LVEDVi) and LV global longitudinal strain (GLS), would be more favorable when compared to isocaloric MICT.

## Materials and Methods

### Study Population

Patients with a first STEMI [diagnosed according to current recommendations (20)] treated by primary PCI within 4 weeks prior to inclusion and who participated in the ambulatory cardiac rehabilitation (CR) program of the Bern University Hospital were recruited for the HIIT-Early study between 30 November 2015 and 30 November 2019.

Exclusion criteria were known chronic heart failure with left ventricular ejection fraction (LVEF) ≤ 45% prior to the acute MI, recent valve surgery, musculoskeletal limitations, thrombus formation, and permanent atrial fibrillation. The study was approved by the ethics committee of the Canton of Bern and registered at ClinicalTrial.gov (NCT02627586). Written informed consent was obtained from all patients.

### Study Design

**FIGURE 1:**
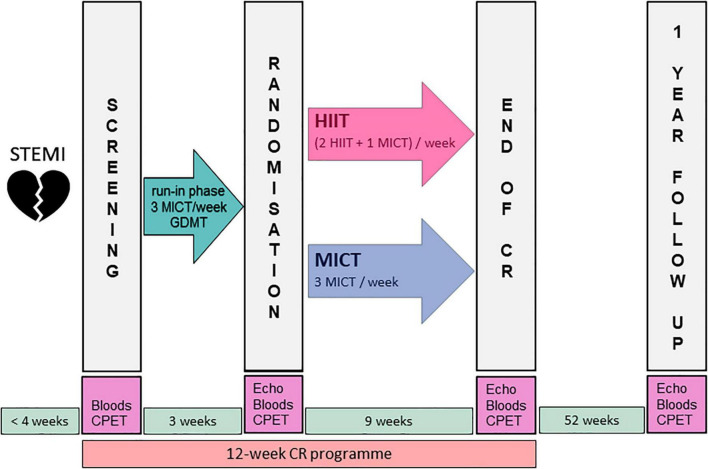
Study flow.

The HIIT-Early study was a single-center, prospective, randomized controlled trial integrated with a 12-week multidisciplinary ambulatory CR program consisting of 36 supervised 90-min exercise training sessions (three per week) including nutrition counseling, psychotherapy, and smoking cessation according to individual needs. The exercise sessions usually included 38 min of endurance training on a cycling ergometer, followed by 45 min of coordination training, resistance training, water therapy, stretching, or relaxation. The exercise intervention has been described in detail elsewhere (18). Briefly, all study participants received a 3-week run-in phase with three weekly MICT sessions and up-titration of GDMT prior to the HIIT or MICT intervention period. During the 9-week intervention period, the MICT group performed 27 MICT sessions, while the HIIT group performed 18 HIIT sessions and nine MICT sessions (2 HIIT and 1 MICT per week). Long-term follow-up was performed 1 year after the conclusion of CR program. The study design is shown in Figure 1. After the run-in phase and after performing the randomization visit, patients were randomly (1:1, blocksize 2) allocated to HIIT or MICT group, stratified by left ventricular function (GLS, cut-off ≤ –12) (21) by research personnel not involved in the intervention delivery using sealed envelopes.

The reporting of the study follows the Consort guidelines for non-pharmacological interventions. Patients were involved in the design of this study by giving feedback on pilot HIIT training.

### Exercise Intervention

#### Run-In Phase

During the run-in phase, all patients underwent the same exercise regime, namely, three MICT sessions per week on a cycle ergometer in order to familiarize patients to exercise training before applying high intensities and for safety reasons. For the first training, exercise intensity was set at the workload of patients’ individual first ventilatory threshold (VT) measured during the cardiopulmonary exercise testing (CPET) of the screening visit. The training sessions lasted 38 min in total, including a 5-min warm-up and 3-min cool-down period at 50% of the training workload. Training intensity was monitored by experienced exercise therapists and adapted weekly based on patients’ perceived exertion, with a target score of 12–13 on the Borg scale (18).

During this run-in period, GDMT was optimized, in particular regarding the dosage of beta-blockers and inhibitors of the renin-angiotensin-aldosterone system.

#### Intervention Phase

Patients randomly allocated to the MICT group continued to perform three MICT training sessions per week, with weekly up-titration to maintain the target Borg scale (18).

Patients allocated to the HIIT group performed two HIIT training sessions interspersed with one MICT training session per week. HIIT consisted of 4 × 4 min intervals with a workload above the second VT (corresponding to approximately 90–95% of peak heart rate), with each interval separated by 3 min of active recovery with a workload below the first VT. The workload of the four high-intensity bouts was kept consistent within each session. Fine-tuning was then performed based on the target Borg scale [as described in detail elsewhere (18)] at the end of the training session to set the workload for the following training session. The total duration of a HIIT session was 38 min, including a 10-min warm-up and 3-min cool-down.

Training workload was adjusted weekly in order to maintain patients’ perceived exertion (Borg 13–14 for MICT, ≥15 for HIIT) and to achieve isocaloric exercise regimens between HIIT and MICT groups (18).

#### Follow-Up Phase: Guideline-Directed Recommendations

During the End-of-CR visit, all patients were given guideline-directed recommendations for physical activity with a minimum of 150 min per week of exercise at moderate to vigorous intensity (22).

### Outcomes

The group difference in LVEDVi at the end of CR analyzed by mixed linear models was the primary outcome. Secondary outcomes were LVEDVi at 1-year follow-up, GLS and other echo parameters, biomarkers of myocardial injury and inflammation, hemodynamic parameters (resting heart and blood pressure), peak VO_2_, and ventilatory efficiency (VE/VCO_2_ slope), at both time points.

### Sample Size Calculation

Based on the absence of previous echocardiographic data with HIIT training in a patient population after recent MI, the sample size calculation was based on a previous study in heart failure patients with a post-intervention LVEDV of 202.9 ± 72.0 ml in the HIIT group and 230.3 ± 41.0 ml in the MICT group (7), achieving an effect size of 0.47. Based on an alpha of 0.05 (one-sided), a power of 80%, and a drop-out rate of 20%, the calculated sample size was 144 patients or 72 in each group (G-Power Version 3.1.9.2, University of Düsseldorf, Germany).

### Blinding

Due to the nature of the intervention, patients were not blinded to the training group allocation. Echocardiographies were performed by experienced sonographers otherwise not involved in the study and blinded to group allocation. They were analyzed off-line by one experienced cardiologist (LDT) who was blinded with regard to patient identity, group allocation, and examination order. CPETs were conducted as clinical routine examinations by the medical staff who were not involved in the study. Therefore, outcome assessors were considered as blinded.

### Data Collection

#### Transthoracic Echocardiographies

Standard transthoracic 2D echocardiography was performed before randomization, at the end of CR, and at the 1-year follow-up. All echocardiographic images were obtained on a Vivid 9 cardiac ultrasound system with a 7.5-MHz transducer (GE Medical Systems, Opfikon, Switzerland) by experienced sonographers of an echo core lab using standard tomographic views. All data were stored on an external hard drive and analyzed offline on a commercially available workstation (EchoPAC, GE Medical Systems, Opfikon, Switzerland).

Traditional echocardiographic parameters of LV geometry, and systolic and diastolic function were assessed based on the most recent recommendations (23, 24). LV internal diameter (LVID), interventricular septum (IVS), and posterior wall thickness (PWT) were measured in M-mode from the parasternal long-axis view at end-diastole. LV mass was calculated based on the cube formula: LV mass = 0.8 × 1.04 × [(IVS + LVID + PWT)3–LVID3] + 0.6 g and indexed for body surface area (BSA) (23). LV end-diastolic (LVEDV) and LV end-systolic volumes (LVESV) were calculated using the biplane method of disks summation technique. Volume measurements were based on tracings of the blood–tissue interface in the apical four- and two-chamber views. At the mitral valve level, the contour was closed by connecting the two opposite sections of the mitral ring with a straight line. LV length was measured from this line to the apical point of the LV contour. All volumes were indexed for BSA. LV systolic function was expressed as ejection fraction (EF), derived from the LVEDV and LVESV. LV diastolic function was assessed by pulse-wave and tissue Doppler in the apical four-chamber view. Peak early and late diastolic velocities at the septal and lateral side of the mitral annulus were recorded and the mean value was calculated and defined as E’ mean (23).

Peak systolic LV GLS was assessed using standard 2D apical four-chamber, two-chamber, and three-chamber view using speckle-tracking analysis (25). All images were recorded during breath-hold over three consecutive cycles using high frame rate loops (50–80 Hz) for reliable analysis by the software. Manual tracing of the endocardial borders on an end-systolic frame (aortic valve closure) was performed, and the myocardial region of interest was adjusted to include all the endocardium and epicardium, excluding the pericardium. Automatic tracing was then applied to the subsequent frames. Adequate tracing for each segment was verified and manually corrected, if necessary. If tracing was still judged incorrect, the specific segment was excluded from the global strain measurement. The global longitudinal strain was determined by averaging all values of the 18 segments of the three views.

#### Biomarkers of Myocardial Injury and Inflammation

Blood samples were obtained in the morning in a fasted state, with the patient in a supine position. We measured the plasma concentrations of high-sensitive troponin T, high-sensitive C-reactive protein (Hs-CRP), and plasma N-terminal (NT) pro-hormone BNP (NT-proBNP) with standard essays from the central laboratory of the Inselspital.

#### Cardiopulmonary Exercise Testing

Cardiopulmonary exercise testing was performed on a cycle ergometer with an individualized ramp protocol aiming to achieve exhaustion within 8–12 min. The protocol consisted of a 3-min warm-up at a workload of 20 Watts followed by an increase of 10, 15, or 20 Watts per minute until voluntary exhaustion and a 2-min active cool-down period. Throughout the CPET, patients were monitored by a cardiologist with continuous assessment of a 12-channel ECG. Breath-by-breath gas exchange was measured using the spirometry system Jaeger Oxycon Pro (Masterscreen CPX, PanGas Healthcare GmbH, Dagmersellen, Switzerland).

Peak VO_2_ was determined as the highest value of a moving average over eight breathing cycles. As a measure of exhaustion, the peak respiratory exchange ratio (RER) was determined by dividing VCO_2_ by VO_2_. We aimed to achieve an RER ≥ 1.10. In addition, the ventilation to carbon dioxide output (VE to CO_2_) slope from ramp start to the respiratory compensation point was derived (26).

#### Training Monitoring

The training workload of the cycling sessions was monitored using the Ers2 system (ergoline GmbH, 72575 Bitz, Germany, Version 1.01). After every training session, patients were asked about the perceived exertion using the established Borg scale (scale of perceived exertion from 6 to 20) (27).

### Data Analysis

We used the R software (R version 3.6.1.) for all statistical analyses.

Patient characteristics were compared between groups using Wilcoxon rank-sum tests, chi-square, or Fisher’s exact test, as appropriate.

Echocardiographic parameters and biomarkers of LV function, hemodynamic parameters, peak VO_2_, and ventilatory efficiency were compared between the two groups by mixed linear models with group and time point as fixed factors and patients as random intercepts, as mixed models can handle missing at random data (28). The group × time interaction for end of CR and 1-year follow-up compared to randomization was assessed. Additional models adjusted for beta-blocker dose and heart rate during echocardiography were performed for LVEDVi and GLS to account for potential confounding.

## Results

### Patients

**FIGURE 2:**
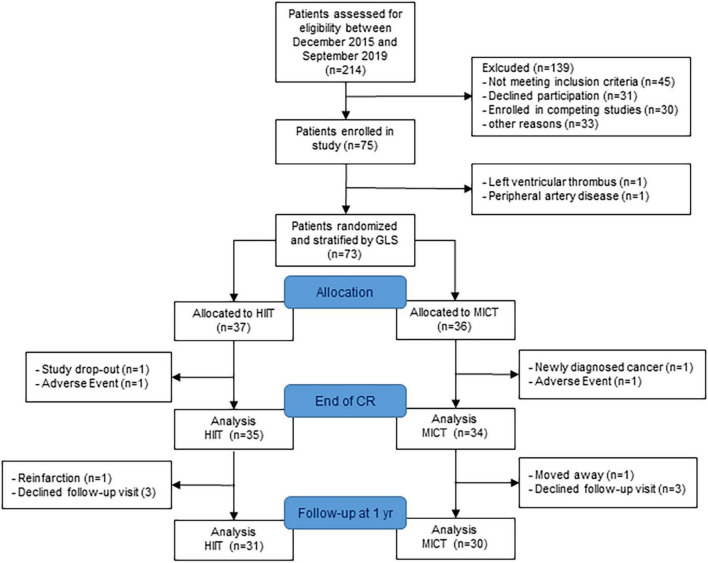
Patient flow. GLS, global longitudinal strain; HIIT, high-intensity interval training; MICT, moderate-intensity continuous training; CR, cardiac rehabilitation.

The study recruitment was stopped after 4 years as planned without reaching the target sample size because of slow recruitment and a further drop in recruitment rate after 3 years due to competing studies. Seventy-five patients were enrolled in the HIIT-Early study, and 69 patients (age 56 ± 10 years) with measurements at randomization and end of CR were finally included in the analysis (35 HIIT and 34 MICT). The patient flow is presented in Figure 2. Patient characteristics at randomization are shown in Table 1. Patients allocated to the HIIT group did not significantly differ from patients allocated to the MICT group. Two patients were lost in each group between randomization and end of CR with one adverse event in each group (5.3% of missing data for primary outcome) and one patient stopping CR and not performing any visits after baseline in the HIIT group and one patient stopping CR and further measurements due to a new cancer diagnosis during CR in the MICT group. Patients of the HIIT group performed a median of 15.3 HIIT sessions (with a median of five additional MICT sessions), while patients of the MICT group performed a median of 22 MICT sessions during the 9-week intervention (18). The detailed results of training characteristics of the two groups have been shown elsewhere (18). Four patients were lost in each group between the end of CR, with three patients in each group performing their 1-year follow-up visit with their private cardiologist. We, therefore, considered missing data as missing at random.

**TABLE 1:** Patient characteristics at randomization.

	MICT, *N* = 34^a^	HIIT, *N* = 35^a^	*P*-value^b^
Age [years]	59 (51, 62)	55 (50, 66)	0.759
Height [cm]	176.7 ± 5.7	174.0 ± 7.2	0.077
Body mass index [kg/m^2^]	26.9 (25.7, 28.9)	26.6 (24.5, 28.8)	0.180
Systolic BP [mmHg]	123 (115, 136)	125 (110, 132)	0.778
Diastolic BP [mmHg]	77 (70, 81)	72 (68, 79)	0.225
**Details of MI**			
Time between MI and screening visit [days]	11 (9, 13)	11 (9, 14)	0.597
Time between MI and randomization [days]	42 (40, 48)	46 (40, 52)	0.51
Culprit lesion			0.99
LAD	17 (50%)	17 (49%)	
LCX	6 (18%)	6 (17%)	
RCA	11 (32%)	12 (34%)	
Number of vessels with hemodynamic relevant stenosis			0.99
1	12 (35%)	13 (37%)	
2	9 (26%)	9 (26%)	
3	13 (38%)	13 (37%)	
**Medication**			
Betablockers (*n*)	33 (97%)	34 (97%)	>0.99
RAAS inhibitors (*n*)	33 (97%)	32 (91%)	0.61
Statins (*n*)	34 (100%)	35 (100%)	1.000
**Cardiovascular risk factors**			
Active smoking	16 (47%)	12 (34%)	0.332
Arterial hypertension	17 (50%)	21 (60%)	0.472
Diabetes mellitus	10 (29%)	7 (20%)	0.413
Dyslipidemia	10 (29%)	12 (34%)	0.797
Family history of CAD	4 (12%)	4 (11%)	1.000
Obesity (BMI > 30 kg/m^2^)	4 (12%)	3 (9%)	0.710

*^a^Shown are median values (IQR); n (%).*

*^b^P-values from Wilcoxon rank-sum test; chi-square test of independence; Fisher’s exact test.*

*BP, blood pressure; HIIT, high-intensity interval training; LAD, left anterior descending; LCX, left circumflex; LVEF, left ventricular ejection fraction; MI, myocardial infarction; MICT, moderate-intensity continuous training; RAAS, blockers of the renin-angiotensin aldosterone system; RCA, right coronary artery; VO_2_, oxygen consumption.*

### Parameters of Left Ventricular Geometry and Function

**FIGURE 3:**
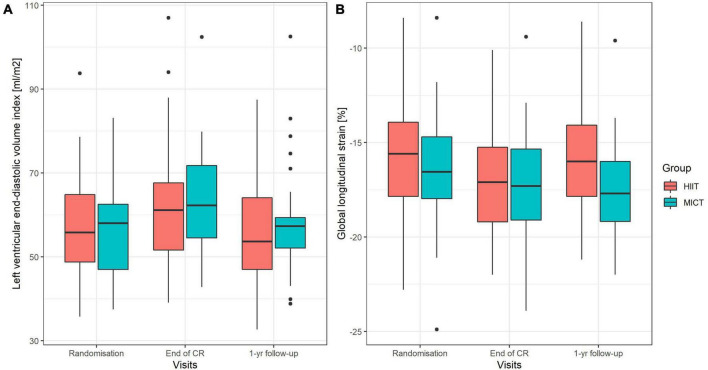
**(A,B)** Boxplots of left ventricular end-diastolic volume index **(A)** and global longitudinal strain **(B)** at randomization, end of CR, and at 1-year follow-up. Boxes include interquartile range (IQR), and the whiskers extend to 1.58 * IQR/√n from the median. HIIT, high-intensity interval training; MICT, moderate-intensity continuous training.

Parameters of LV geometry and function measured during the three visits are shown for the two groups separately in Table 2. Results of the models for the main parameters of LV function, namely, LVEDVi and GLS, are shown in Table 3. LVEDVi increased significantly (*p* = 0.003) from randomization to end of CR by 4.5 ml/m^2^ (95% CI: 0.5 to 8.4 ml/m^2^) in HIIT and 6.2 ml/m^2^ (2.1 to 10.2 ml/m^2^) in MICT, with no significant difference between the groups. At 1-year follow-up, LVEDVi was not significantly different compared to randomization in HIIT and MICT, changing by –1.9 ml/m^2^ (95% CI: –6.1 to 2.3 ml/m^2^) and 1.3 ml/m^2^ (–2.9 to 5.5 ml/m^2^), respectively (Figure 3A). Comparable results were obtained with the model adjusted for beta-blocker dose and heart rate (Table 3). For GLS, values improved non-significantly (*p* = 0.081) between randomization and end of CR in HIIT by –0.9% (–1.7 to –0.1%) and MICT by –0.7% (–1.6 to 0.9%, Table 3) without significant group × time interaction. From randomization to 1-year follow-up, there was a significant (*p* = 0.031) group × time interaction for GLS, changing by –1.0% (–1.8 to –0.1%) in the MICT group and by 0.3% (–0.5 to 1.2%) in the HIIT group (Figure 3B). The group × time interaction effect in GLS from randomization to 1-year follow-up remained significant in the model adjusted for heart rate and beta-blocker dose (Table 3). Unadjusted mean ± SD changes between randomization and 1-year follow-up were 0.61 ± 2.93% in the HIIT and –0.95 ± 2.05% in the MICT group, resulting in a Cohen’s d effect size of 0.60 (pooled SD 2.61). The change over time in LVEDVi and GLS of individual patients is shown in Supplementary Figure 1.

**TABLE 2:** Left ventricular echocardiography parameters, biomarkers, and cardiorespiratory fitness at randomization, end of CR, and 1-year follow-up.

	HIIT *n* = 35 Median [IQR]			MICT *n* = 34 Median [IQR]			*P*-values Interaction group*time^a^	
			
	Randomization	End of CR	1-Year follow-up	Randomization	End of CR	1-Year follow-up	End of CR	1-Year follow-up
LV EDV index [ml/m^2^]	55.8 [48.8; 64.8]	61.1 [51.6; 67.6]	53.7 [47.0; 64.1]	58.0 [47.0; 62.5]	62.3 [54.5; 71.8]	57.3 [52.1; 59.4]	0.557	0.297
LV ESV index [ml/m^2^]	25.2 [17.5; 30.7]^1^	25.0 [20.7; 32.1]	24.1 [16.7; 29.7]^6^	21.9 [17.5; 28.2]	24.2 [20.5; 29.0]^2^	22.5 [18.7; 29.2]^5^	0.609	0.388
GLS [%]	–15.6 [–17.9; –13.9]	–17.1 [–19.2; –15.3]	–16.0 [–17.9; –14.1]	–16.6 [–18.0; –14.7]	–17.3 [–19.1; –15.4]	–17.7 [–19.2; –14.1]	0.825	0.031
LV EF [%]	57.0 [49.4; 64.4]	56.2 [53.3; 62.3]	57.0 [53.4; 62.8]^4^	61.1 [52.7; 65.5]	62.1 [56.7; 65.0]^1^	58.3 [54.4; 62.5]^4^	0.915	0.766
LV mass index [g/m^2^]	115 [93; 134]^1^	104 [89; 128]^3^	96 [75; 116]^4^	101 [79; 125]^1^	106 [97; 123]^3^	110 [94; 126]^6^	0.489	0.015
E/A ratio	0.89 [0.76; 1.22]	0.98 [0.81; 1.17]	0.89 [0.81; 1.16]^4^	0.93 [0.80; 1.20]	0.99 [0.86; 1.27]^1^	0.94 [0.76; 1.10]^3^	0.246	0.635
E’ mean	7.80 [6.58; 9.78]	8.35 [7.00; 9.90]^2^	8.50 [7.20; 10.59]^5^	9.00 [7.85; 10.00]^1^	9.40 [7.63; 10.20]^3^	8.90 [6.68; 10.30]^3^	0.562	0.069
E/E’ mean	8.56 [6.96; 9.68]	7.74 [6.79; 10.19]	7.44 [6.54; 8.57]^5^	7.75 [6.58; 9.13]	7.93 [6.82; 9.40]^1^	8.05 [6.23; 9.43]^3^	0.192	0.074
Heart rate [bpm]	60.5 [57.0; 67.0]^1^	60.0 [54.0; 67.0]^2^	63.0 [57.0; 69.0]^6^	62.0 [56.3; 70.3]	59.5 [54.5; 63.3]^2^	63.0 [57.0; 70.0]^5^	0.780	0.470
Systolic BP [mmHg]	125 [110; 132]	124 [115; 135]	130 [120; 140]^6^	123 [9115; 136]	122 [115; 128]^1^	127 [121; 133]^3^	0.143	0.077
Diastolic BP [mmHg]	72 [68; 79]	76 [64; 81]	80 [73; 85]^6^	77 [70;81]	71 [66; 79]^1^	77 [70; 83]^3^	0.022	0.003
Hs-Troponin T [ng/l]^b^	14.0 [10.5; 26.0]^8^	10.0 [6.5; 15.5]^12^	6.0 [5.0; 9.8]^9^	12.0 [8.8; 16.5]^6^	9.0 [6.8; 10.3]^10^	7.0 [6.0; 8.5]^7^	0.474	0.079
NT-proBNP [pg/ml]^b^	424 [153; 903]^2^	184 [89; 381]^3^	118 [70; 177]^8^	210 [127; 547]	99 [58; 247]^3^	68 [38; 157]^5^	0.813	0.781
Hs-CRP [mg/l]^b^	1.24 [0.65; 2.39]^4^	0.90 [0.50; 2.54]^1^	0.86 [0.47; 1.39]^9^	0.99 [0.65; 1.64]	0.82 [0.48; 1.30]	0.75 [0.46; 1.53]^7^	0.686	0.347
Peak VO_2_ [ml/kg/min]	26.5 [24.4; 31.1]	29.6 [25.3; 32.2]	29.0 [26.6; 33.3]^6^	27.7 [23.9; 31.6]	29.9 [26.1; 34.9]	30.6 [26.0; 33.8]^4^	0.138	0.317
VE/VCO_2_ slope	32.9 [29.7; 35.1]	30.0 [29.0; 33.9]	30.0 [27.0; 33.3]^6^	31.5 [27.9; 33.9]	30.0 [27.2; 32.0]	30.4 [28.0; 33.0]^4^	0.225	0.205

*^a^P-values for interaction terms of group × time point (with reference randomization) from mixed models with patients as random intercepts.*

*^b^Mixed linear models for biomarkers of myocardial damage were performed by taking the natural logarithm of the biomarker values due to non-normal distribution of these values.*

*Superscript numbers indicate number of missing values. HIIT, high-intensity interval training; MICT, moderate-intensity continuous training; IQR, interquartile range; LV, left ventricle; EF, ejection fraction; ESV, end-systolic volume; E, peak transmitral flow velocity in early diastole; E’ mean, mean velocity of early diastolic mitral annular motion as determined by pulsed wave Doppler; A, peak transmitral flow velocity in late diastole; BP, blood pressure; Hs, high sensitivity; NT-proBNP, N-terminal pro-b-type natriuretic peptide; VO_2_, oxygen consumption; VE, ventilation; CO_2_, carbon dioxide output; HR, heart rate.*

**TABLE 3:** Mixed linear models for left ventricular end-diastolic volume index and global longitudinal strain with patients as random intercepts.

Dependent variable	Independent variable	Model 1			Model 2		
			
		Estimate	SE	*p*-value	Estimate	SE	*P*-value
LV EDVi [ml/m^2^]	Intercept	57.13	2.30	0.000	67.00	7.28	0.000
	End-of CR	6.15	2.05	0.003	6.04	2.10	0.005
	1-year follow-up	1.26	2.12	0.554	2.53	2.22	0.258
	HIIT	–0.00	3.24	0.999	–0.47	3.19	0.884
	Betablocker [%]				0.06	0.05	0.246
	Heart rate				–0.18	0.10	0.088
	HIIT* End-of CR	–1.69	2.86	0.557	–2.41	2.86	0.403
	HIIT*1-year follow-up	–3.16	3.01	0.297	–0.87	3.23	0.788
GLS [%]	Intercept	–16.47	0.53	0.000	–20.15	1.58	0.000
	End-of CR	–0.73	0.42	0.081	–0.34	0.45	0.452
	1-year follow-up	–0.95	0.42	0.026	–0.82	0.47	0.080
	HIIT	0.46	0.74	0.538	0.36	0.71	0.612
	Betablocker [%]				0.02	0.01	0.052
	Heart rate				0.05	0.02	0.030
	HIIT*End-of CR	–0.13	0.58	0.825	–0.45	0.60	0.455
	HIIT*1-year follow-up	1.30	0.60	0.031	1.55	0.66	0.021

*Model 1 includes fixed factors group (with MICT as reference) and time point (with randomization as reference) as well as their interaction terms. Model 2 additionally includes fixed factors beta-blocker dose of maximal dose and heart rate.*

*SE, standard error; LV EDVi, left ventricular end-diastolic volume indexed to body surface area; GLS, global longitudinal strain; HIIT, high-intensity interval training; MICT, moderate-intensity continuous training.*

### Hemodynamic Response

There was no change over time and no between-group differences in heart rate during echocardiography from randomization to 1-year follow-up (Table 2). There was an increase in systolic BP of 7.9 mmHg (1.5–14.3 mmHg) in the HIIT group and a decrease of 0.2 mmHg (–6.4 to 6.1 mmHg) in the MICT group (*p*-value for interaction 0.077). Diastolic BP decreased by 1.3 mmHg (–5.2 to 2.7 mmHg) in the MICT group but increased by 7.3 mmHg (3.3 to 11.4 mmHg) in the HIIT group (*p*-value for interaction 0.003). This resulted in a Cohen’s d effect size between groups of 0.44 for systolic BP change and 0.61 for diastolic BP change.

### Biomarkers of Myocardial Injury and Inflammation

Hs-Troponin T was missing in 8-12 patients of the HIIT group, depending on the visit, and 6–10 patients of the MICT group (Table 3). NT-proBNP was missing in 2–8 HIIT patients and 0–5 MICT patients, and Hs-CRP in 1–9 HIIT patients and 0–7 MICT patients. There were no significant group × time interactions for any of the parameters (Table 2).

### Cardiorespiratory Response

Mean peak VO_2_ improved significantly from randomization to 1-year follow-up by 2.4 ml/kg/min (95% CI: 1.0 to 3.7 ml/kg/min) in HIIT and by 3.4 ml/kg/min (2.0 to 4.7 ml/kg/min) in MICT patients, with no significant group × time interaction (Table 2). Likewise, comparable improvements for both groups were observed for the VE to CO_2_ slope, with –2.2 (–3.3 to –1.0) for HIIT and –1.1 (–2.2 to 0.0) for MICT (Table 2). Detailed results of cardiorespiratory parameters in this cohort and verification of isocaloric HIIT (median 5.1, interquartile range 4.6–5.9 METs) and MICT (5.2, 4.4–5.9 METs) have been published elsewhere (18).

## Discussion

The main findings of our randomized controlled trial were as follows: (1) in optimally treated patients early after STEMI, short- and long-term effects of HIIT on LVEDVi, cardiorespiratory fitness, and biomarkers of myocardial injury and inflammation were not different compared to isocaloric MICT, and (2) a median 15.2 (interquartile range 13.6–16.6) HIIT sessions had less favorable long-term effects on GLS compared to MICT sessions only. This is a novel finding that deserves further investigation. Noteworthy, all patients were treated with primary PCI, and the majority had a normal LV ejection fraction at the time of randomization.

### Left Ventricular Geometry and Function

In our cohort of post-STEMI patients with nearly normalized LVEF at randomization due to the short onset-to-balloon time at our tertiary referral center, we found comparable increases of approximately 6 and 4 ml/m^2^ in LVEDVi over the 9-week intervention period in the MICT and HIIT groups, respectively, which were no longer different to randomization levels at 1-year follow-up (Table 2). This is in contrast to earlier studies in patients after acute MI. A small pilot study of eight patients after acute MI who performed HIIT over a 12-week training period and 10 patients who received physical activity recommendations only found no changes in LVEDVi in either group (17). Likewise, the SAINTEX-CAD study did not find any changes in LVEDVi after neither a 12-week HIIT nor MICT training. However, the SAINTEX-CAD study included a heterogeneous population of 115 patients 4–12 weeks after acute MI, 25 patients after PCI, and 60 patients after coronary bypass grafting (CABG) (10). Further, a smaller study including 34 patients after MI, PCI, or CABG, who employed a combination of HIIT and MICT training, found no changes in LVEDVi over the 12-week training program (29). This is in line with an older study including 149 patients who started a more moderate 3-month exercise training program within 10–14 days after MI with moderately to severely reduced LVEF where LV end-diastolic diameter was also not increased after training (30). Another study in 30 patients after acute MI with moderately reduced LVEF even found LVEDVi to be significantly reduced after a moderate 6-month exercise training program, while it was increased in the 30 control patients without exercise (31, 32). In a recent multi-center study in clinically stable heart failure patients with reduced ejection fraction (EF), both MICT and HIIT have been shown to increase EF (33) and reduce LVED diameter (8). At 1-year follow-up, LVED diameter was found to be reduced similarly with HIIT and MICT in stable heart failure patients (8). We suggest that in our cohort with normal to mildly dilated LV (except for one patient with moderate dilatation) (34), the increase in LVEDVi from randomization to end of CR in both groups may reflect a normal exercise-induced training adaptation. This is supported by the fact that the change in LVEDVi over this time period was neither related to NT-proBNP levels at randomization nor change in NT-proBNP over this time period, rather it was inversely related to LVEDVi values at randomization and resting heart rate, which is likely to reflect an increase in stroke volume.

In the present study, no significant time effect or group × time interaction was found in GLS from before to after the 9-week intervention period. However, at 1-year follow-up, GLS improved significantly in the MICT group (–1%, *p* = 0.026) but deteriorated by 0.4% in the HIIT group, leading to a significant time × group interaction (*p* = 0.031, Table 3, Model 1, Figure 3B). The effects of HIIT and MICT training on GLS was measured only in two previous studies. Similar to our findings, Trachsel et al. (17) found no changes in GLS over the 12-week HIIT training period in their pilot study in a comparable population. Likewise, in the SAINTEX-CAD study, no changes over the 3-month training period were found for GLS in neither the MICT nor HIIT group in stable CAD patients (10). The clinical relevance of this novel finding remains unclear and deserves further investigation. Interestingly, HIIT resulted in higher systolic and in particular diastolic BP at 1-year follow-up, compared to MICT (however, results of models did not change when BPs were entered into the models). This is in accordance with findings of a recent meta-analysis that found consistently lower systolic and diastolic BP after MICT than HIIT training (35). Whether this is causally related to the decline in GLS observed in the HIIT group remains to be established (36). A possible explanation for higher BP values after HIIT could be increased sympathetic activity. We demonstrated acute and chronic differences in heart rate and its variability between HIIT and MICT in our cohort compatible with this hypothesis (37).

While studies comparing HIIT and MICT after recent MI are sparse in humans, there have been animal studies that looked at the potentially damaging effects of intense exercise. One study implemented either 4 or 8 weeks of HIIT and MICT in mice and found higher collagen accumulation in the myocardium and higher serum inflammation markers in the HIIT compared to the MICT group (38). Further, infarct size was reduced by a greater amount in the MICT than in the HIIT group (38). Another study implemented 4 weeks of either training form and found that the mitochondrial number of cardiomyocytes increased with both training forms, but mitochondria in the HIIT mice were swollen and vacuolated with disrupted cristae, while this was not the case in the MICT mice (39). An adverse change in GLS has been shown to be the consequence of myocardial fibrosis in hypertensive rats (40). The association between myocardial fibrosis and GLS has also been found in human studies (41).

### Cardiorespiratory Response

Our study is in line with previous studies comparing isocaloric HIIT and MICT protocols, as classified by the meta-analysis of Gomes-Neto et al. (6). We did not find any significant differences between HIIT and MICT with regard to changes in peak VO_2_. In fact, we even observed a trend toward greater improvements in MICT (+3.2 ml/kg/min) compared to HIIT (+1.9 ml/kg/min) patients (*p* = 0.104) (18). Comparable results were reported by Trachsel et al. (16) who found a greater proportion of peak VO_2_ responders in MICT compared to HIIT patients. Comparable peak VO_2_ improvements in patients performing either MICT or HIIT (both + 20.3%) over an intervention period of 12 weeks (36 sessions) were found in the largest study to date comparing HIIT with MICT in patients with CAD (58% after acute myocardial infarction), namely the SAINTEX-CAD study, which included 200 patients (13, 42). There were, however, many studies that have found greater increases in peak VO_2_ with HIIT compared to MICT in patients with CAD (43) and cardiovascular disease (44).

### Clinical Perspective

Our study adds to the evidence that both in chronic and acute coronary syndrome patients, HIIT is not superior to isocaloric MICT with regard to LV remodeling (10, 17) and peak VO_2_ (13, 42). We, therefore, advocate that patients may choose the exercise intensity according to their own preference. This is likely to improve long-term adherence. Our results of adverse long-term changes in GLS after HIIT warrant caution with recommending vigorous activities in the early phase after an acute STEMI.

### Strengths

A major strength of our study was the homogenous cohort of STEMI patients enrolled within 4 weeks after primary PCI. The randomized study design with a run-in period, which facilitated the optimization of GDMT and provided a familiarization test (baseline testing), was another obvious strength of our study. HIIT was introduced only after the 3-week exercise training familiarization period with three weekly MICT sessions, and during the intervention period, only two HIIT sessions per week were performed, interspersed by one weekly MICT session, which corresponds to a feasible low-dose HIIT program (18). It is unlikely that a higher dose of HIIT would have resulted in more favorable LV remodeling, rather, effects of a higher dose of HIIT may have an even more adverse effect on GLS in patients early after acute STEMI.

### Limitations

Due to too many concomitantly running competing studies, the number of included patients did not achieve the target sample size of 144. However, based on our results, to achieve a significant time × group interaction for LVEDVi at end of CR, we would have needed 3,164 patients, illustrating the negligible clinical effect. Whether fewer patients would have been needed if patients performed three HIIT sessions per week rather than two plus one MICT session remains open for investigation. In contrast, the significant deterioration of GLS in the HIIT group detected at 1-year follow-up even in this small sample was an unexpected but probably clinically relevant finding. Our results apply to male STEMI patients only, as we did not include any female patients. The proportion of female patients treated with STEMI at our center was 23% within the last 10 years. They had a lower uptake of CR, higher mean age, and more comorbidities (45). Thus, we expected a low number of female eligible patients and excluded them in order to avoid additional heterogeneity. Lastly, we cannot exclude that engagement in physical activity during and after CR differed between groups, as we did not measure this.

In optimally treated patients early after acute STEMI, HIIT was not different from isocaloric MICT with regard to short- and long-term effects on LVEDVi and cardiorespiratory fitness. The worsening in GLS at 1 year in the HIIT group is a novel finding that deserves further investigation. It may be connected to the higher blood pressure found in the HIIT group by us and others (35).

## Data Availability Statement

The raw data supporting the conclusions of this article will be made available by the authors, without undue reservation.

## Ethics Statement

The studies involving human participants were reviewed and approved by Ethics committee of the Canton of Berne. The patients/participants provided their written informed consent to participate in this study.

## Author Contributions

MW, J-PS, and LT designed the study. LT, AZ, and MW recruited the patients and determined their individual training zones. LT, IF, TM, SD, and DH were involved in data collection. PE, TM, IF, and DH processed the data and performed the data analyses. PE wrote the first draft of the manuscript. All authors approved the final version of the manuscript.

## Conflict of Interest

The authors declare that the research was conducted in the absence of any commercial or financial relationships that could be construed as a potential conflict of interest.

## Publisher’s Note

All claims expressed in this article are solely those of the authors and do not necessarily represent those of their affiliated organizations, or those of the publisher, the editors and the reviewers. Any product that may be evaluated in this article, or claim that may be made by its manufacturer, is not guaranteed or endorsed by the publisher.
