# Supplementary figures and images for: Short- and Long-Term Effects of High-Intensity Interval Training vs. Moderate-Intensity Continuous Training on Left Ventricular Remodeling in Patients Early After ST-Segment Elevation Myocardial Infarction—The HIIT-EARLY Randomized Controlled Trial
Source: Front Cardiovasc Med. 2022 Jun 17;9:869501. doi: 10.3389/fcvm.2022.869501 (PMC9247394; doi:10.3389/fcvm.2022.869501)

Supplement Figure 1a and b

a)

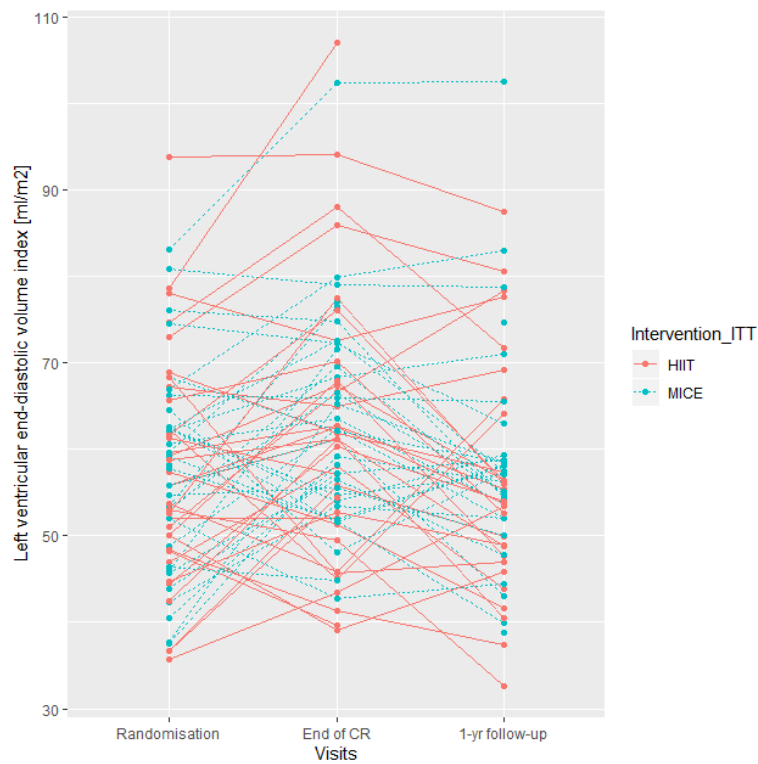

b)

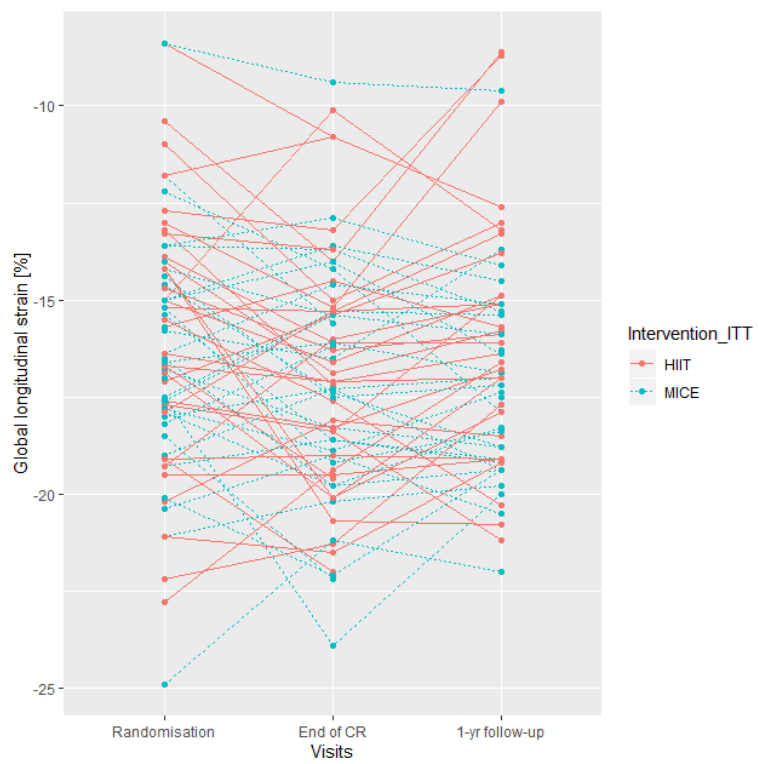

Supplement: Supplementary file 1 [file Image_1.pdf]
